# Development of a quantitative job exposure matrix for standing, walking, and forward bending among pregnant workers – The PRECISE JEM

**DOI:** 10.5271/sjweh.4252

**Published:** 2025-11-01

**Authors:** Hannah Nørtoft Frankel, Esben Meulengracht Flachs, Camilla Sandal Sejbaek, Jonathan Aavang Petersen, Jens Peter Bonde, Ingrid Sivesind Mehlum, Mette Korshøj, Susan Peters, Magnus Svartengren, Pasan Hettiarachchi, Peter J Johansson, Alex Burdorf, Luise Mølenberg Begtrup

**Affiliations:** 1 Department of Occupational and Environmental Medicine, Copenhagen University Hospital - Bispebjerg and Frederiksberg, Copenhagen, Denmark.; 2 Department of Public Health, University of Copenhagen, Copenhagen, Denmark.; 3 National Institute of Occupational Heath (STAMI), Oslo, Norway.; 4 Department of Occupational and Social Medicine, Copenhagen University Hospital Holbæk, Denmark.; 5 Environmental Epidemiology Division, Institute for Risk Assessment Sciences, Utrecht University, Utrecht, The Netherlands.; 6 Occupational and Environmental Medicine, Department of Medical Sciences, Uppsala University, Uppsala, Sweden.; 7 Occupational and Environmental Medicine, Uppsala University Hospital, Uppsala, Sweden.; 8 Department of Public Health, Erasmus MC, Rotterdam, The Netherlands.

**Keywords:** adverse pregnancy outcome, exposure assessment, linear mixed-effects model, occupational physical activity

## Abstract

**Objectives:**

Occupational physical activity (OPA) during pregnancy has been linked to adverse pregnancy outcomes, but crude exposure assessment remains an issue in causal inference. We aimed to develop a quantitative trimester-specific job exposure matrix (JEM) for standing, walking, and forward bending among pregnant workers.

**Methods:**

Accelerometer measurements from 403 female workers across 109 DISCO-08 job codes were obtained in Denmark between January 2023 and June 2024. Full workdays were measured during two weeks among pregnant workers and one week among non-pregnant workers. We used linear mixed-effects models to estimate exposure levels of occupational standing, walking, and forward bending for all 1171 DISCO-08 codes, including age, trimester, and expert ratings as fixed effects, and job codes and workers as random effects.

**Results:**

The between-job variances relative to total variances were 56% for standing, 51% for walking, and 45% for forward bending. The fixed effect trimester reduced standing time by 0.38 hours during the 3^rd^ trimester compared to non-pregnant participants, whereas no differences were observed for walking or forward bending. Based on the *trimester-specific JEM* for occupational standing time, bakers had the highest exposure (range from non-pregnant to 3^rd^ trimester, 5.41–5.03 hours/workday). For walking and forward bending, the highest exposed jobs from the *pregnancy-specific JEM* were waiters (1.76 hours/workday) and livestock/dairy producers (1.24 hours/workday), respectively.

**Conclusions:**

The JEM enhances independent objective exposure assessment in epidemiological studies of OPA and pregnancy outcomes and may advance guidelines and potentially prevent adverse pregnancy outcomes.

Occupational physical activities (OPA) during pregnancy, including standing, walking, and forward bending, have been identified as potential risk factors for several adverse pregnancy outcomes such as miscarriage, preterm birth, and fetal growth restriction, although results are inconsistent (1–5).

In Denmark, approximately 80% of women of reproductive age are active in the labor force (6). A large Danish survey found that approximately 50% of women aged 18–45 years spend at least three-quarters of their workday standing or walking, and 40% worked in strenuous postures with their back twisted for at least a quarter of their workday (7). As most workers continue their employment throughout pregnancy, many remain exposed to various OPA throughout pregnancy.

Exposure assessment of OPA remains a significant challenge, particularly in large-scale studies, which can be managed by utilizing job exposure matrices (JEM) (8, 9). A JEM is a cross-tabulation of jobs and estimated exposure levels, establishing a correspondence between occupations and specific exposure estimates. Once developed, JEM enable reproducible and cost-effective analysis of exposure-response relationships in epidemiological studies. Further, using JEM helps minimize information bias when compared to self-reported exposure assessments (8, 9).

Most prior studies investigating the associations between OPA and adverse pregnancy outcomes have relied on self-reported exposure data, which carries a risk of information bias, and in some studies, recall bias as well (10–17). Some studies have utilized JEM, however, these JEM were based on self-reported data (18–21) or expert assessment only (22–25), potentially limiting their accuracy (26). Additionally, most existing JEM were not developed for pregnant workers, which is a limitation, given potential differences in OPA and increased vulnerability during pregnancy. The few existing pregnancy-specific JEM are questionnaire-based (20) or expert-based (23, 25), and assess exposure at a single time point, failing to capture potential variations across pregnancy stages, thus reducing accuracy.

JEM developed with repeated technical measurements provide more detailed and objective information and may therefore aid in establishing more reliable guidelines (27). Therefore, we aimed to develop a quantitative, trimester-specific JEM for occupational standing, walking, and forward bending ≥30° for pregnant workers, combining individual technical measurements with expert ratings.

## Methods

### Data collection and selection

Between January 2022 and June 2024, pregnant workers were recruited to the PRECISE Occupational Cohort at the 1^st^ trimester ultrasound scan (pregnancy week 11–13) from six obstetric departments in Denmark. Inclusion criteria were age ≥18 years, singleton pregnancy, being in paid employment, and English/Danish speaking. The recruitment process is described in detail elsewhere (28). Briefly, a subgroup from the cohort was chosen from an a priori list of specified occupations selected by both a high prevalence of female workers and a broad range of expected OPA to ensure adequate coverage of occupations held by pregnant workers in Denmark (29). This subgroup was invited to wear accelerometers continuously for one week during both the 2^nd^ and 3^rd^ trimesters. Due to the time of recruitment (pregnancy week 11–13), participation in the 1^st^ trimester was not possible. Therefore, non-pregnant female workers aged 18–45 years, mainly coworkers of the pregnant participants, were invited to participate for one week and served as a proxy for 1^st^ trimester.

All participants answered a questionnaire at the time of inclusion, including job title, and participants were assigned DISCO-08 (30) [the Danish-2008 version of the International Standard Classification of Occupations, ISCO-2008 (31)] job codes by the occupational specialist LMB. Job titles were confirmed during a telephone conversation.

### Measurements of occupational physical activities

Occupational time spent standing, walking, and forward bending ≥30° was measured with two synchronized tri-axial accelerometers, Axivity AX3® (Axivity Ltd, Newcastle upon Tyne, UK), placed on the mid-right thigh and the upper back (T1-T2 level) (28). Participants were asked to wear the accelerometers continuously for seven days and register work time, sleep time, and any non-wear time in a provided diary. Data were processed with the customized software ActiPASS (github.com/Ergo-Tools/ActiPASS) based on the validated activity detection algorithm Acti4 (32–34). Participant diaries were entered in ActiPASS to differentiate work time, and data patterns were scrutinized through individual quality-check visualizations and checked for outliers with, eg, high trunk angles. Occupational time spent standing and walking (hours/workday) was determined using ActiPASS algorithms from thigh-worn accelerometers, while forward bending ≥30°(hours/workday) was derived from trunk-worn accelerometers, recorded in one-second epochs. Standing time was defined as a standing posture with intermittent steps. Walking time was defined as purposeful walking. Forward bending was defined as time spent with forward trunk inclination ≥30° while in a standing position, where the reference angle was calculated from the participants’ mean trunk angle during walking.

Measurements were considered valid if they comprised ≥8 work hours/participant, and each workday consisted of ≥4 work hours (35). Each participant’s workday was duration-normalized to 8-hour time-weighted averages (TWA).

### Expert assessment

*Occupational group strategy.* The DISCO-08 is a five-level (six-digit) hierarchical code structure, classifying and aggregating jobs (30). The first four levels are nearly identical to ISCO-08 (31) whereas the most detailed level five (consisting of six digits) is a further division of labor functions in Denmark. At level five, there are 563 groups comprising 1171 job codes. Utilizing the hierarchical structure, two occupational specialists (LMB and CB) grouped 1050 of the 1171 job codes into 350 groups. This was done to the extent where all exposures assessed (standing, walking, forward bending ≥30°) were expected to be equal within all jobs in the group, and with respect to the hierarchical structure of DISCO-08. Eg, exposures within level five ‘226610 Work in audiology’ and ‘226620 Work in speech therapy’ were considered equal and grouped to the less detailed level four ‘2266 Work within audiology and speech therapy’.

### Benchmarks

To qualify the expert ratings, two authors a priori chose 33 occupational groups to be assessed for consensus by the experts. Occupational groups were chosen for benchmarks by expected broad variations in OPA, and by having information derived from at least two of the following three estimates/measurements: (i) sitting time and standing/walking time from the “Lower Body JEM” (36), (ii) sitting time from the JEM developed from the Work Environment and Health in Denmark cohort study (37) restricted to females aged 18–45 years, (iii) standing, walking, sitting, and forward bending ≥30° mean measurements from the PRECISE Occupational Cohort (28) with at least five subjects in each occupational group.

### Expert ratings

Three experts, two professors and one specialist in occupational medicine, all experienced with JEM, rated all occupational groups according to a 1^st^ trimester pregnant worker in a regular 8-hour work shift and noted if they expected exposure reduction throughout pregnancy. The experts met and discussed each of the 33 benchmark occupational groups until consensus for each exposure was reached. Thereafter, all three raters independently rated the remaining 317 (350–33) occupational groups.

Occupational time spent standing, walking, and sitting were each rated from 1–5 (1=very little, 2=about a quarter, 3=about half, 4=about three-quarters or 5=almost all the time) with the constraint to sum their own three ratings to 7. Forward bending ≥30°, while in upright position, was rated with categories 1–4 (1=0–15, 2=16–30 minutes, 3=31–60 or 4=>60 minutes) in line with a large Norwegian survey (38). It was also possible to state 0=cannot be assessed. The semi-quantitative categorical expert ratings were hereafter converted to fully quantitative measures (hours per 8-hour workday) by fitting best linear models, as done in a similar study (39).

### Imputations of expert ratings

DISCO-08 codes that did not receive expert ratings due to rating at higher levels (N=121) were assigned the mean of each expert’s assessments from the corresponding higher-level codes. For instance, level three ‘513 Waiters and bartenders’ was not originally assigned an expert rating due to ratings at the more detailed level four ‘5131 Waiters’ and ‘5132 Bartenders’. Imputations were provided by the averages of the corresponding codes, in this example, the mean of ‘5131 Waiters’ and ‘5132 Bartenders’ from each expert.

### Statistical methods

To construct the JEM, we analyzed variance components and estimated predictors of exposures by fitting linear mixed-effects models (lme4-package in R) using restricted maximum likelihood estimation following the method in Peters et al (40, 41). Job (DISCO-08 code) and worker (participant) were included as random effects, and age, trimester, and expert ratings were included as fixed effects. Body mass index (BMI), parity, previous miscarriages, and whether the experts expected a reduction in OPA throughout pregnancy did not improve the model fit or improve explanatory power for any of the three exposures and were therefore not included in the final models. Education is closely linked to the DISCO-08 system, and therefore accounted for by the random effect job and not included as a fixed effect due to high correlation with job code. With this approach, expert ratings could only affect between-job variance, while age could influence both between-job and between-worker variance, and trimester could impact all three variance components: between-job, between-worker, and within-worker variance.

The measured exposure levels of standing, walking, and forward bending ≥30° were included as 8-hour TWA, calculated per workday and per participant, and served as the dependent variables.

The random effect terms for job and worker were assumed statistically independent and normally distributed with mean zero and provided two different variance components representing the between-job variance and the between-worker (within-job) variance, respectively. We obtained best linear unbiased predictions (BLUP) of the random effect for each of the jobs with exposure measurements (N=109). The BLUP adjusted the estimates by shrinking them toward the overall mean in jobs where measurements were limited, and pulled the estimates closer to the individual measurements in jobs with many measurements. We aimed at assessing exposure levels of standing, walking, and forward bending ≥30° for all 1171 DISCO-08 codes. Jobs without measurements were assigned model-driven exposure level estimates from the corresponding expert rating. Exposure estimates for jobs with available measurements (N=109) incorporated the job-specific prediction by the statistical model (BLUP). Derived model outcomes were utilized to estimate the eight-hour TWA of each exposure across all DISCO-08 codes.

Due to a general overestimation of expert assessment of walking time compared to the technical measurements (supplementary material, www.sjweh.fi/article/4252, figure S2b), the square root of the expert walking time was used for the statistical modeling. Model adequacy was assessed by confirming normal distribution of residuals using Q-Q plots, as well as testing for linearity and homoscedasticity through scatterplots of residuals against fitted values. All analyses were performed using R version 4.2.2.

## Results

**Figure 1:**
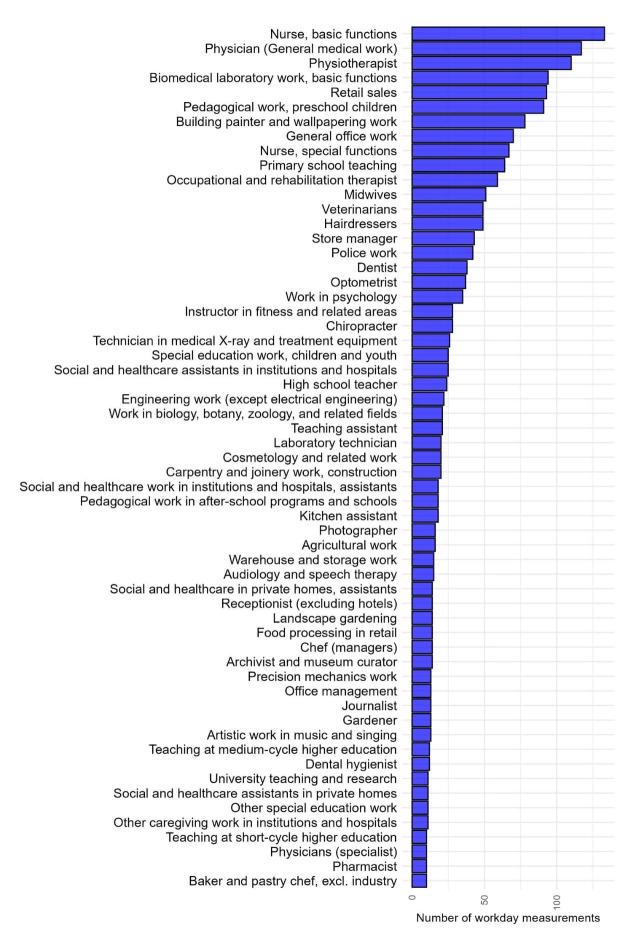
Distribution of workday accelerometer measurements (N=1973) across job titles with at least ten measurements each (N=59).

In total, we obtained accelerometer measurements from 403 participants, providing a total of 553 weeklong measurements (83 non-pregnant participants, 309 during 2^nd^ and 161 during 3^rd^ trimester) comprising 2224 workday measurements. Distribution of workday measurements across the most frequent jobs is depicted in figure 1.

Median age was 29.6 years and 73% had at least three years of higher education at the time of inclusion. Median hours/week of measured work time was 31.0 (5^th^, 95^th^ percentiles 13.0, 45.7) and median daily work time was 7.2 hours (5^th^, 95^th^ percentiles 4.3, 9.4) (table 1).

**Table 1:** Participant characteristics (N=403) and accelerometer-based activity measures.

		N ^a^	%	Mean	Median ^b^	5^th^ / 95^th^ percentiles ^b^
Age (years)				30.9	29.6	24.6 / 38.6
Body mass index ^c^				25.0	23.8	19.1 / 35.7
	Missing	3	1			
Parity ^d^						
	0	186	58			
	1	104	33			
	≥2	30	9			
Previous miscarriage(s) ^d^		76	24			
Highest educational level						
	Primary school	9	2			
	Vocational education	47	12			
	High school/business school	15	4			
	Short tertiary education (≤2.5 years)	34	8			
	Medium tertiary education (3–4 years)	182	45			
	Long tertiary education (≥5 years)	113	28			
	Missing	3	1			
Accelerometer-based measurements ^e^						
	Weekly work time measured (hours)			30.4	31.0	13.0 / 45.7
	Daily work time measured (hours)			7.1	7.2	4.3 / 9.4
Pregnancy week at 2^nd^ trimester measurement				18.1	17.7	14.1 / 23.6
Pregnancy week at 3^rd^ trimester measurement				29.6	29.4	27.4 / 32.3
Dimensions from accelerometers ^f^						
	Standing time (hours) ^g^			2.7	2.6	0.7 / 5.4
	Walking time (hours) ^g^			0.8	0.7	0.2 / 1.7
	Sitting time (hours) ^g^			4.5	4.6	1.3 / 7.0
	Forward bending ≥30° (hours) ^h^			0.5	0.4	0.1 / 1.2

^a^ 403 unique participants: 83 non-pregnant and 320 pregnant (309 with measurements in the 2^nd^ trimester and 161 in the 3^rd^ trimester). ^b^ Medians, 5^th^ and 95^th^ percentiles were based on information from five individuals with values closest to the actual median/percentile. ^c^ BMI (kg/m^2^) was pre-pregnancy for pregnant participants and current BMI for non-pregnant participants. ^d^ Information not available from non-pregnant participants. ^e^ Measurements were per participant and trimester. ^f^ Dimensions from accelerometers were calculated as 8-hour time-weighted averages per workday and per participant. ^g^ No missing data from thigh accelerometers. ^h^ Missing data from trunk accelerometers was N=25 (6%).

In the linear mixed-effects null-models, which included only random effects (job and worker), approximately half of the total variance was attributed to differences between jobs (56% for standing, 51% for walking, and 45% for forward bending ≥30°) (table 2). Introducing age and trimester as fixed effects (model 1) reduced between-job variance by 2%, between-worker variance by 3%, and within-worker variance by 1% for standing. Age and trimester did not distinctly influence the between-job or between-worker variances for walking or forward bending ≥30°. When including expert ratings (final model), we observed a 50% reduction of between-job variance for both standing and forward bending ≥30°, and 17% for walking, compared to the null-model.

**Table 2:** Variance components for standing, walking, and forward bending ≥30° derived from the linear mixed-effects models.

Variance components		Null-model ^a^			Model 1 ^b^				Final model ^c^		
		Variance	%		Variance	%	Percentage reduction ^d^		Variance	%	Percentage reduction ^d^
Standing ^e^											
	Between jobs	1.33	56		1.31	56	2		0.67	39	50
	Between workers	0.46	19		0.45	19	3		0.45	26	3
	Within worker	0.60	25		0.60	25	1		0.60	35	1
	Total	2.39	100		2.35	100	2		1.71	100	28
Walking ^e^											
	Between jobs	0.13	51		0.13	51	0		0.11	46	17
	Between workers	0.06	23		0.06	23	0		0.06	25	0
	Within worker	0.07	26		0.07	26	0		0.07	29	0
	Total	0.26	100		0.26	100	0		0.23	100	9
Forward bending ≥30° ^f^											
	Between jobs	0.07	45		0.07	45	1		0.04	29	50
	Between workers	0.05	31		0.05	31	0		0.05	40	0
	Within worker	0.04	24		0.04	24	0		0.04	31	0
	Total	0.16	100		0.16	100	0		0.13	100	22

^a^ The null model includes job and subject ID as random effects. ^b^ Model 1 includes, in addition to the null model, age and trimester as fixed effects. ^c^ The final model includes, in addition to model 1, expert ratings as fixed effects. ^d^ Percentage reduction of variance explained by fixed effects when compared with the null model. ^e^ The variance components were based on 2224 observations from 403 unique participants and 109 occupational codes (DISCO-08). ^f^ Variance components were based on 2083 observations from 389 unique participants and 107 occupational codes (DISCO-08).

Time spent standing decreased minimally with increasing age, with an annual decline of 0.02 hours (1.2 minutes) (table 3). A reduction in standing time was observed in the 3^rd^ trimester, 0.38 hours (23 minutes) compared to non-pregnant participants. No significant differences for time spent walking or forward bending ≥30° were seen with increasing age or trimesters.

**Table 3:** Fixed effect model parameters from the final linear mixed-effects model for standing, walking, and forward bending ≥30°. [CI=confidence interval]

			Standing ^a^			Walking ^a^			Forward bending ≥ 30° ^b^	
			β (hours)	95% CI		β (hours)	95% CI		β (hours)	95% CI
Intercept			2.228	1.50– 0.99		0.496	0.20–0.81		0.309	0.08–0.53
Expert ratings			0.472	0.36–0.58		0.334	0.18–0.48		0.598	0.44–0.76
Age			-0.021	-0.04–0.00 ^c^		-0.002	-0.01–0.01		-0.003	-0.01–0.00
Trimester										
	Non-pregnant		Ref.			Ref.			Ref.	
		2^nd^	-0.203	-0.41–0.00		0.028	-0.04–0.10		-0.036	-0.10–0.03
		3^rd^	-0.380	-0.59– -0.17		-0.006	-0.08–0.07		-0.057	-0.12–0.01

^a^ The fixed effects model parameters were based on 2224 observations from 403 unique participants and 109 occupational codes (DISCO-08). ^b^ The fixed effects model parameters were based on 2083 observations from 389 unique participants and 107 occupational codes (DISCO-08). ^c^ The 95% confidence interval was rounded to 0.00. P-value was 0.040.

For each increased hour from the expert ratings, there was an increase of 0.47 hours (28 minutes) of standing time, 0.33 hours (20 minutes) of walking time, and 0.59 hours (35 minutes) of forward bending ≥30°, indicating positive relationships between expert ratings and each exposure.

When restricting the analyses to jobs with ≥10 participants in each job code (N=185), we observed in addition to a reduction in standing time in third trimester compared to non-pregnant participants of 0.44 hours (26 minutes), also a reduction in time spent forward bending ≥30° of 0.11 hours (7 minutes), but no significant reduction for time spent walking (supplementary tables S1 and S2). When restricting the analyses to pregnant participants with measurements in both 2^nd^ and 3^rd^ trimesters (N=150), we saw a 5% reduction in between-worker variances for walking and forward bending when adjusting for age and trimester (model 1) (supplementary table S3). Further, there were reductions in all three exposures for age and for the 3^rd^ trimester compared to the 2^nd^ trimester, although only significant for standing and walking (supplementary table S4).

Model-based estimates from the *trimester-specific* standing JEM found that the highest exposed job for occupational standing was bakers, pastry-cooks, and confectionery makers (range from non-pregnant to 3^rd^ trimester was 5.41–5.03 hours). The *pregnancy-specific* JEM estimates for the highest exposed jobs for walking and forward bending ≥30° were waiters (1.76 hours) and livestock/dairy producers (1.24 hours), respectively (table 4 and supplementary table S5). Exposure differences between the highest and lowest exposed jobs were approximately fivefold for standing and walking, and eightfold for forward bending ≥30°.

**Table 4:** Model-based mean exposure levels for the three highest and lowest exposed jobs at the four-digit DISCO-08 level (the Danish 2008 version of the International Standard Classification of Occupations).

**The three highest-exposed jobs for standing time**		**Mean standing time range ^a^ (hours/8-hour workday)**
7512	Bakers, Pastry-cooks and Confectionery Makers	5.41–5.03
5223	Shop Sales Assistants	5.19–4.81
7131	Painters and Related Workers	4.95–4.57
**The three lowest-exposed jobs for standing time**		
8342	Earthmoving and Related Plant Operators	1.12–0.74
1431	Sports, Recreation and Cultural Centre Managers	1.26–0.88
1323	Construction Managers	1.48–1.10
**The three highest-exposed jobs for walking time**		**Mean walking time (hours/8-hour workday)**
5131	Waiters	1.76
5246	Food Service Counter Attendants	1.61
9121	Hand Launderers and Pressers	1.60
**The three lowest-exposed jobs for walking time**		
2261	Dentists	0.31
8342	Earthmoving and Related Plant Operators	0.39
4321	Stock Clerks	0.41
**The three highest-exposed jobs for forward ** **bending ≥30 °**		**Mean forward bending ≥30 ° time (hours/8-hour workday)**
6121	Livestock and Dairy Producers	1.24
5131	Waiters	1.00
5164	Pet Groomers and Animal Care Workers	0.88
**The three lowest-exposed jobs for forward bending ≥30 °**		
4226	Receptionists (general)	0.15
8342	Earthmoving and Related Plant Operators	0.17
1431	Sports, Recreation and Cultural Centre Managers	0.17

^a^ Mean standing time range time covers the mean from non-pregnant participants to the mean for 3^rd^ trimester participants. Walking and forward bending ≥30 ° times are not trimester-specific.

Comparison of our PRECISE JEM estimates for standing and walking to the general population Lower Body JEM (38) revealed an overall ratio of 0.75 (supplementary table S6), indicating lower exposures during pregnancy, with no systematic deviation over exposure mean (supplementary figure S1).

Inter-class correlations (ICC) between the three expert assessments for all 350 occupational groups after benchmark consensus were between 0.82–0.88 (supplementary table S7), corresponding to ‘good agreement’ between raters for all three exposures (42). Mirror plots with comparisons of expert ratings with accelerometer measurements for standing, walking, and forward bending ≥30° are depicted in supplementary figure S2.

## Discussion

We have developed a quantitative JEM for standing, walking, and forward bending ≥30° for pregnant workers based on technical measurements and expert ratings, which is, to the best of our knowledge, the first of its kind.

In the linear mixed-effects null-models, we found that most of the variance was between jobs (56% for standing, 51% for walking, and 45% for forward bending ≥30°). These high between-job variances are comparable to the few other quantitative JEM based on both measurements and expert ratings, including the recently published quantitative JEM for solar ultraviolet radiation (43) wood dust (44) and noise (45) and even higher between-job variances than in the JEM for daytime light exposure (46) and carcinogenic agents (41, 47). A high between-job variance is an important feature, as it represents the occupational-specific differences in exposure of question and is therefore a necessity to investigate associations in epidemiological studies when applying JEM.

### Methodological considerations

We hypothesized a reduction in all three exposures throughout pregnancy, however, we only observed a reduction in occupational standing time, which was approximately 17% (0.38/2.22). The reduction in exposures was likely attributable to physiological changes during pregnancy, including rapid weight gain and increased abdominal circumference, which may impair work ability. Psychological factors and workplace accommodations may also contribute. The reason we did not find a reduction in walking and bending may be caused by insufficient power, indicated by the stronger reduction effect of trimester in the sensitivity analyses, including only jobs with ≥10 workers (supplementarytable S2) and only pregnant participants with measurements in both the 2^nd^ and 3^rd^ trimesters (supplementary table S4). Further, if reduction only occurs in some jobs but not others, the model’s ability to detect an overall effect would be weakened. Another possible reason is that occupational standing time might be more easily replaced with sitting than tasks that require walking or bending. Finally, our results are to some extent subject to healthy worker effect, as many workers in the 3^rd^ trimester were on sick leave, which may have affected the results.

Due to our model results, the JEM for standing has been made trimester-specific, whereas the JEM for walking and forward bending ≥30° are merely pregnancy-specific. Measurements across trimesters and in more jobs are needed and may result in even more detailed JEM in the future.

### Strengths and limitations

The newly developed PRECISE JEM has several strengths. Firstly, the JEM is based on repeated, technical measurements from mainly pregnant workers.

The use of repeated measurements has been demonstrated to increase precision (48). We included repeated measurements from all workers, both by repeated workdays within a work week and repeated measurements for many of the pregnant workers during both the 2^nd^ and 3^rd^ trimesters. This contributed to improving precision in the mixed-effects models by accounting for within-worker variability, and it enabled investigation of exposure variations throughout trimesters, thus strengthening the JEM. Pregnancy-related discomforts, whether related to work or not, and workplace adaptations may contribute to a reduction in OPA throughout pregnancy, which was confirmed by the observed decrease in standing time in the 2^nd^ and 3^rd^ trimesters compared to non-pregnant workers. The trimester-specific standing JEM will therefore enable novel investigation of OPA and pregnancy outcomes, while taking potential changes of exposure throughout pregnancy trimesters into account.

Another strength of the JEM is the usage of Axivity accelerometers and the customized software, ActiPASS, with the validated Acti4 algorithm providing high accuracy of the assessments of standing, walking, and forward bending ≥30° (32, 49, 50). Technical measurements are often regarded as more reliable than self-reported methods (51, 52).

The overall high agreements between the three experts in the assessment for standing, walking, and forward bending ≥30° strengthened the JEM (42). We also observed an overall high correlation when comparing accelerometer measurements and expert ratings for standing and forward bending ≥30°, however, we did observe a general overestimation of expert ratings for walking time (supplementary figure S2). When comparing expert-assessed and inclinometer-measured upper arm elevation >90°, Dalbøge et al (53) found similar overestimation by expert ratings. In contrast, Korshøj et al (54) revealed good correspondence comparing expert ratings with accelerometer measurements for the combination of standing and walking within 16 job titles. The discrepancies we observed between expert ratings and accelerometer measurements for walking time may result from the challenge facing experts to accurately assess walking and standing time separately. Kember et al (55) highlighted this issue and recommended wearables to distinguish postures like standing and walking. To adjust for the discrepancy of walking time, we used the square root of the expert assessment walking time in the mixed-effects model. Nevertheless, the results from the final model, which included expert ratings, revealed a lower reduction of between-job variance for walking (17%) than standing and forward bending ≥30° (50%), when compared to the null-model, indicating that the expert ratings explained less of the variance. Thus, walking time estimates from our JEM may be subject to greater uncertainty.

Finally, a strength of the JEM was the reduced risk of misclassification of job title, as participants were asked to write their job title as specifically as possible, and job titles were confirmed during telephone calls. When in doubt, participants were asked to further describe their job function for appropriate coding.

A limitation of the PRECISE JEM is the application of a uniform exposure reduction adjustment for trimesters across all occupations, potentially overlooking occupation-specific differences. The experts noted whether they expected reduction in exposure throughout pregnancy for each occupational group. However, when we included this reduction term in the model, it had no significant impact on the estimates, leading to its exclusion.

Another limitation of this study includes the lack of measurements from the 1^st^ trimester, which was due to the study design of the PRECISE Occupational Cohort, where recruitment took place at the beginning of the 2^nd^ trimester. Using measurements from non-pregnant coworkers as a proxy might have caused inaccuracies. Weight gain or other physical changes are limited during the 1^st^ trimester, and we would not expect substantial differences in, eg, ability to stand, walk, or bend forward. However, other factors could change physical work patterns, such as nausea and fatigue, and therefore, the estimates of 1^st^ trimester exposure should be used with more caution. Finally, a limitation is normalizing workdays to 8 hours, which may reduce precision; however, sensitivity analyses using weekly averages showed similar results (data not shown), supporting the validity of this approach.

### Comparison to previous findings

To our knowledge, this is the first quantitative JEM for standing, walking, and forward bending ≥30° based on the combination of technical measurements and expert ratings, and also the first among pregnant workers. Previous studies using JEM to investigate the association between OPA and adverse pregnancy outcomes have mostly utilized *non-pregnancy-specific* JEM (18, 19, 21, 22, 24). These studies may overlook associations due to non-differential misclassification if physical work conditions are modified for pregnant workers, and thresholds for adverse effects could be lower than detected. Comparison of PRECSE JEM estimates and the general population Lower Body JEM (36), confirmed lower exposures during pregnancy (supplementary table S6, figure S1). Despite methodological differences in the JEM, the discrepancies reinforce the need for a pregnancy-specific JEM. The few previous studies investigating the association between OPA and adverse pregnancy outcomes with JEM that were specifically developed for pregnant workers (20, 23, 25) have other drawbacks. Mocevic et al (20) developed their JEM for occupational lifting using a single questionnaire at median pregnancy week 16, potentially underestimating any true effect of OPA if exposure decreases throughout pregnancy – a noted limitation. To our knowledge, no previous JEM have considered exposure differences throughout pregnancy. The other two pregnancy-specific JEM were solely based on expert ratings (23, 25) and were limited to a small number of occupations (25), providing crude estimates and limited possibility to use them in large populations.

### Generalizability

The PRECISE Occupational Cohort consisted of a higher proportion of highly educated participants compared to the general population (28, 56). This is a common problem in birth cohorts (57) and is reflected in the jobs held by the participants. The PRECISE JEM had measurements from roughly 20% of all six-digit DISCO-08 codes. While many jobs were well represented, including many of the most frequent jobs held by the female population (figure 1) (56) many jobs relied heavily on expert ratings, and more measurements in rare but higher exposed jobs would strengthen the JEM.

Measurements were reduced to a smaller number of workers in the 3^rd^ trimester, primarily due to pregnancy-related absence. Consequently, the data collected, especially during the 3^rd^ trimester, may represent healthier workers and less physically demanding jobs compared to the general pregnant working population. Future studies applying this JEM should account for pregnancy-related absence to prevent differential misclassification. When accounting for actual work time during pregnancy, it is also important to address the risk of healthy worker selection bias; otherwise, associations may be biased towards the null. Healthy worker selection bias must be considered when developing guidelines based on such studies. Lower cut-off values than those suggested by future studies based on our JEM could be necessary to ensure the protection of pregnant workers, who might be more susceptible but were underrepresented in the study population.

While JEM provide standardized estimates, individual circumstances matter. In Denmark, there are recommendations limiting standing and walking from the fourth pregnancy month, but not forward bending (58). Although JEM cannot assess individual compliance, such guidelines likely influence job-level estimates. Future studies should apply the PRECISE JEM in populations comparable to the Danish workforce, preferably among pregnant workers, to ensure its appropriate use and validity.

### Concluding remarks

We have developed a quantitative trimester-specific JEM for standing and a pregnancy-specific JEM for walking and forward bending ≥30°. The PRECISE JEM is a valuable tool for further investigation of OPA and adverse pregnancy outcomes in large-scale epidemiological studies. Use of more accurate exposure assessment may contribute to improve guidelines for pregnant workers and potentially help prevent adverse pregnancy outcomes.

## Supplementary material

Supplementary material

## Protection of research participants

The Knowledge Centre on Data Protection Compliance approved the study (Privacy: P-2022-201), which the Scientific Ethics Committee deemed exempt. It was registered at ClinicalTrials.gov (NCT05670145) prior to inclusion and complies with the Declaration of Helsinki.

## Declarations

Generative artificial intelligence (ChatGPT) was used to translate job titles from Danish to English and for a language check of the manuscript. The first author thoroughly reviewed and edited the content as necessary and assumes full responsibility for the final published article.
