# Supplementary material for: Development of a quantitative job exposure matrix for standing, walking, and forward bending among pregnant workers – The PRECISE JEM
Source: Scand J Work Environ Health. 2025 Oct 30;51(6):526–36. doi: 10.5271/sjweh.4252 (PMC12593706; doi:10.5271/sjweh.4252)
Supplement: Supplementary material [file SJWEH-51-526-S001.pdf]

Development of a quantitative job exposure matrix for standing, walking, and forward bending among pregnant workers – The PRECISE JEM[1]

by Hannah Nørtoft Frankel, MD,[2] *Esben Meulengracht Flachs, PhD, Camilla Sandal Sejbaek, PhD, Jonathan Aavang Petersen, PhD, Jens Peter Bonde, DSc, Ingrid Sivesind Mehlum, PhD, Mette Korshøj, PhD, Susan Peters, PhD, Magnus Svartengren, PhD, Pasan Hettiarachchi, PhD, Peter J Johansson, PhD, Alex Burdorf, PhD, Luise Mølenberg Begtrup, PhD*

1. Supplementary Material
2. Correspondence to: Hannah Nørtoft Frankel, Department of Occupational and Environmental Medicine, Copenhagen University Hospital - Bispebjerg and Frederiksberg, Copenhagen, Denmark. [E-mail: hannah.noertoft.frankel.01@regionh.dk]

Table S1. Variance components for standing, walking and forward bending ≥30° derived from the sensitivity linear mixed-effects models for jobs with at least ten participants in each job code (DISCO-08).

**Variance components**	**Null model**[a]		**Model 1**[b]			**Final model**[c]		
	Variance	%	Variance	%	Percentage reduction [d]	Variance	%	Percentage reduction[d]
**Standing**[e]								
Between jobs	0.908	50	0.900	50	1	0.467	35	49
Between workers	0.353	19	0.338	19	4	0.338	25	4
Within worker	0.551	30	0.545	31	1	0.545	40	1
Total	1.812	100	1.783	100	2	1.351	100	25
**Walking**[e]								
Between jobs	0.048	30	0.047	30	2	0.051	32	-6
Between workers	0.044	28	0.043	28	1	0.043	27	1
Within worker	0.065	42	0.065	42	0	0.065	41	0
Total	0.157	100	0.156	100	1	0.159	100	-1
**Forward bending ≥ 30°** [f]								
Between jobs	0.034	34	0.035	35	-1	0.013	17	61
Between workers	0.032	32	0.030	31	5	0.030	39	5
Within worker	0.034	34	0.033	34	1	0.033	44	1
Total	0.100	100	0.098	100	2	0.077	100	23

[a] The null model includes job and subject ID as random effects.
[b] The model 1 includes, in addition to the null model, age and trimester as fixed effects.
[c] The final model includes, in addition to model 1, expert ratings as fixed effects.
[d] Percentage reduction of variance explained by fixed effects when compared with the null model.
[e] The variance components were based on 1026 observations from 185 unique participants and 12 occupational codes (DISCO-08).
[f] The variance components were based on 964 observations from 179 unique participants and 12 occupational codes (DISCO-08).

Table S2. Fixed effect model parameters from the linear mixed-effects model analyses for the sensitivity analyses, restricted to jobs with at least ten participants in each job code (DISCO-08). [CI=confidence interval]

	Standing[a]		Walking[a]		Forward bending ≥ 30° [b]	
	β(hours)	95%CI	β(hours)	95%CI	β(hours)	95%CI
**Intercept**	1.978	(0.81 – 3.14)	0.941	(0.46 – 1.42)	0.408	(0.14 – 0.68)
**Expert ratings**	0.405	(0.16 – 0.65)	0.088	(-0.22 – 0.40)	0.539	(0.28 – 0.80)
**Age**	-0.017	(-0.04 – 0.01)	-0.007	(-0.01 – 0.00)	-0.007	(-0.01 – 0.00)[c]
**Trimester**						
Non-pregnant	Ref		Ref		Ref	
2nd	-0.251	(-0.50 – 0.00)	-0.020	(-0.11 – 0.07)	-0.072	(-0.14 – 0.00)
3rd	-0.439	(-0.70 – -0.18)	-0.049	(-0.14 – 0.04)	-0.113	(-0.19 – -0.04)

[a] The fixed effects model parameters were based on 1026 observations from 185 unique participants and 12 occupational codes (DISCO-08).

[b] The fixed effects model parameters were based on 964 observations from 179 unique participants and 12 occupational codes (DISCO-08).

[c] The confidence interval was rounded to 0.00. p-value was 0.03.

Table S3. Variance components for standing, walking, and forward bending ≥30° derived from the sensitivity linear mixed-effects models including only pregnant participants with measurements during both the 2nd and 3rd trimesters (n= 150).

Variance components	Null-model[a]		Model 1[b]			Final model[c]		
	Variance	%	Variance	%	Percentage reduction *	Variance	%	Percentage reduction[d]
**Standing**[e]								
Between jobs	1.091	53	1.043	52	4	0.650	40	40
Between workers	0.354	17	0.350	17	1	0.355	22	0
Within worker	0.629	30	0.621	31	1	0.620	38	1
Total	2.074	100	2.014	100	3	1.626	100	22
**Walking**[e]								
Between jobs	0.079	41	0.075	40	6	0.063	36	21
Between workers	0.044	23	0.042	22	5	0.041	24	5
Within worker	0.070	36	0.070	37	0	0.070	40	0
Total	0.193	100	0.186	100	4	0.174	100	10
**Forward bending ≥30°** [f]								
Between jobs	0.070	46	0.069	46	2	0.039	32	45
Between workers	0.042	28	0.040	27	5	0.041	34	1
Within worker	0.040	26	0.040	27	0	0.040	33	0
Total	0.152	100	0.149	100	2	0.120	100	21

[a] The null model includes job and subject ID as random effects.
[b] Mhe model 1 includes, in addition to the null model, age and trimester as fixed effects.
[c] The final model includes, in addition to model 1, expert ratings as fixed effects.
[d] Percentage reduction of variance explained by fixed effects when compared with the null model.
[e] The variance components were based on 1207 observations from 150 unique participants and 65 occupational codes (DISCO-08).
[f] The variance components were based on 1127 observations from 149 unique participants and 65 occupational codes (DISCO-08).

Table S4. Fixed effect model parameters from the sensitivity linear mixed-effects model analyses, including only pregnant participants with measurements during both the 2nd and 3rd trimesters (n=150). [CI=confidence interval]

	Standing[a]		Walking[a]		Forward bending ≥ 30° [b]	
	**β(hours)**	**95%CI**	**β(hours)**	**95%CI**	**β(hours)**	**95%CI**
**Intercept**	2.412	(1.35 – 3.49)	0.943	(0.56 – 1.33)	0.490	(0.16 – 0.82)
**Expert ratings**	0.390	(0.24 – 0.54)	0.254	(0.09 – 0.42)	0.555	(0.34 – 0.77)
**Age**	-0.028	(-0.06 – 0.00)	-0.014	(-0.02 – 0.00)[c]	-0.009	(-0.02 – 0.00)
**Trimester**						
2nd	Ref.		Ref.		Ref.	
3rd	-0.181	(-0.27 – -0.09)	-0.031	(-0.06 – 0.00)[d]	-0.023	(-0.05 – 0.00)

[a] The fixed effects model parameters were based on 1207 observations from 150 unique participants and 65 occupational codes (DISCO-08).

[b] The fixed effects model parameters were based on 1127 observations from 149 unique participants and 65 occupational codes (DISCO-08).

[c] The confidence interval was rounded to 0.00. p-value was 0.006.

[d] The confidence interval was rounded to 0.00. p-value was 0.046.

Table S5. Model-based mean exposure levels for the ten highest and lowest exposed jobs at the four-digit DISCO-08 level (the Danish 2008 version of the International Standard Classification of Occupations).

The ten highest-exposed jobs for standing time		Mean standing time range[a] (hours/8-hour workday)
7512	Bakers, Pastry-cooks and Confectionery Makers	5.41-5.03
5223	Shop Sales Assistants	5.19-4.81
7131	Painters and Related Workers	4.95-4.57
7132	Spray Painters and Varnishers	4.95-4.57
7511	Butchers, Fishmongers and Related Food Preparers	4.95-4.57
7522	Cabinet-makers and Related Workers	4.95-4.57
8121	Metal Processing Plant Operators	4.90-4.52
5120	Cooks	4.73-4.35
7114	Concrete Placers, Concrete Finishers and Related Workers	4.69-4.31
7119	Building Frame and Related Trades Workers Not Elsewhere Classified	4.69-4.31
**The ten lowest-exposed jobs for standing time**		
8342	Earthmoving and Related Plant Operators	1.12-0.74
1431	Sports, Recreation and Cultural Centre Managers	1.26-0.88
1323	Construction Managers	1.48-1.10
5412	Police Officers	1.58-1.20
2211	Generalist Medical Practitioners	1.71-1.33
2634	Psychologists	1.74-1.36
8311	Locomotive Engine Drivers	1.78-1.40
2266	Audiologists and Speech Therapists	1.82-1.44
1111	Legislators	1.82-1.45
1112	Senior Government Officials	1.82-1.45
**The ten highest-exposed jobs for walking time**		**Mean walking time (hours/8-hour workday)**
5131	Waiters	1.76
5246	Food Service Counter Attendants	1.61
9121	Hand Launderers and Pressers	1.60
6130	Mixed Crop and Animal Producers	1.46
6121	Livestock and Dairy Producers	1.43
5120	Cooks	1.42
3423	Fitness and Recreation Instructors and Programme Leaders	1.32
9611	Garbage and Recycling Collectors	1.31
3434	Head chefs	1.31
3432	Interior Designers and Decorators	1.28
**The ten lowest-exposed jobs for walking time**		

2261	Dentists	0.31
8342	Earthmoving and Related Plant Operators	0.39
4321	Stock Clerks	0.41
5412	Police Officers	0.42
2651	Visual Artists	0.43
2267	Optometrists and Ophthalmic Opticians	0.46
4226	Receptionists (general)	0.48
2266	Audiologists and Speech Therapists	0.49
7314	Potters and Related Workers	0.50
2310	University and Higher Education Teachers	0.51
**The ten highest-exposed jobs for forward bending ≥ 30 °**		**Mean forward bending ≥ 30 ° time (hours/8-hour workday)**
6121	Livestock and Dairy Producers	1.24
5131	Waiters	1.00
5164	Pet Groomers and Animal Care Workers	0.88
9121	Hand Launderers and Pressers	0.85
6113	Gardeners	0.85
7112	Bricklayers and Related Workers	0.85
7115	Carpenters and Joiners	0.85
7119	Building Frame and Related Trades Workers Not Elsewhere Classified	0.85
7121	Roofers	0.85
7122	Floor Layers and Tile Setters	0.85
**The ten lowest-exposed jobs for forward bending ≥ 30 °**		
4226	Receptionists (general)	0.15
8342	Earthmoving and Related Plant Operators	0.17
1431	Sports, Recreation and Cultural Centre Managers	0.17
5412	Police Officers	0.18
8311	Locomotive Engine Drivers	0.18
2310	University and Higher Education Teachers	0.20
1323	Construction Managers	0.20
4110	General Office Clerks	0.20
0110	Commissioned Armed Forces Officers	0.20
1111	Legislators	0.21

[a] Mean standing time range time covers the mean for non-pregnant participants to the mean for 3rd trimester participants. Walking and forward bending ≥ 30 ° times are not trimester-specific.

Table S6. Comparison of standing and walking JEM estimates between The PRECISE JEM and general population Lower Body JEM

DISCO-08	Title	PRECISE JEM estimates (the sum of standing and walking, 2nd trimester)	Lower Body JEM estimates, standing/walking	Ratio (PRECISE estimate/Lower Body JEM estimate)
132300	Management of core activities in construction	1.90	2.60	0.73
216300	Work with product and fashion design	2.62	6.10	0.43
221100	General medical work	2.01	4.30	0.47
222110	Nursing work, basic functions	3.80	5.00	0.76
222200	Midwifery work	2.88	5.00	0.58
225000	Veterinary work	3.65	4.20	0.87
265200	Artistic work within music and singing	3.35	5.80	0.58
311400	Technician work within the electronic field	2.65	4.75	0.56
324000	Assisting veterinary work and veterinary technician work	4.24	4.20	1.01
332200	Sales work (agents)	2.37	2.80	0.85
343200	Interior designer and decorator work	4.52	6.10	0.74
343500	Other work in artistic, cultural, and culinary fields	3.24	6.10	0.53
432100	Warehouse dispatch work	2.09	5.22	0.40
511120	Passenger service in airports and harbour terminals	4.43	6.00	0.74
512000	Cook work	5.95	6.60	0.90
513100	Waiter work	6.07	6.60	0.92
514100	Hairdresser work	4.97	5.50	0.90
514200	Cosmetologist work and related functions	3.38	5.50	0.61
522300	Sales work in stores	6.19	6.01	1.03
532120	Social and health work in institutions and hospitals, assistants	3.80	5.44	0.70
532190	Other care work in institutions and hospitals	2.61	5.44	0.48
532210	Social and health work in private homes, helpers	3.60	5.44	0.66
611310	Production and greenhouse gardening work	5.60	5.88	0.95
611320	Landscaping work	3.44	5.88	0.58
611390	Other gardening work	4.20	5.88	0.71
612100	Work with breeding of livestock, excluding poultry	4.47	4.80	0.93
613000	Work with plant cultivation and animal breeding	4.80	4.80	1.00
711510	Carpentry and joinery work, building	5.40	5.80	0.93
713110	Building painter and wallpapering work	4.50	5.96	0.75
722100	Blacksmithing work	4.72	6.00	0.79
751200	Bakery and confectionery work, excluding industry	6.35	7.30	0.87
812100	Operator work in metal processing	5.71	7.00	0.82
815900	Other operator work in the production of textile, fur, and leather products	3.22	5.72	0.56
831100	Locomotive driver work	2.22	4.60	0.48
833210	Truck drivers, national transport	2.56	1.65	1.55
834200	Construction machinery operator work	1.31	3.25	0.40

911210	Office and residential area cleaning	4.11	6.51	0.63
911220	Hospital cleaning, etc.	4.00	6.51	0.61
912100	Manual washing and pressing of clothes	6.01	6.30	0.95
933410	Warehouse and depot work	4.31	5.03	0.86
941210	Assistance in the kitchen	6.23	6.51	0.96
				Mean Ratio: 0.75

[a] DISCO-08 jobs were restricted to jobs that overlapped between the two JEMs and are frequently held by female workers (selected by jobs measured in the PRECISE Cohort).

[b] The Lower Body JEM(38).

Table S7. Inter Class Correlations (ICC) between the three experts for assessments in each exposure (standing, walking, forward bending ≥30°) for all 350 occupational groups after benchmark consensus. [CI=confidence interval]

**Expert exposure assessment**	**ICC**	**95% CI**
Standing	0.88	(0.85 - 0.90)
Walking	0.82	(0.78 - 0.85)
Forward bending ≥30°	0.87	(0.84 - 0.89)

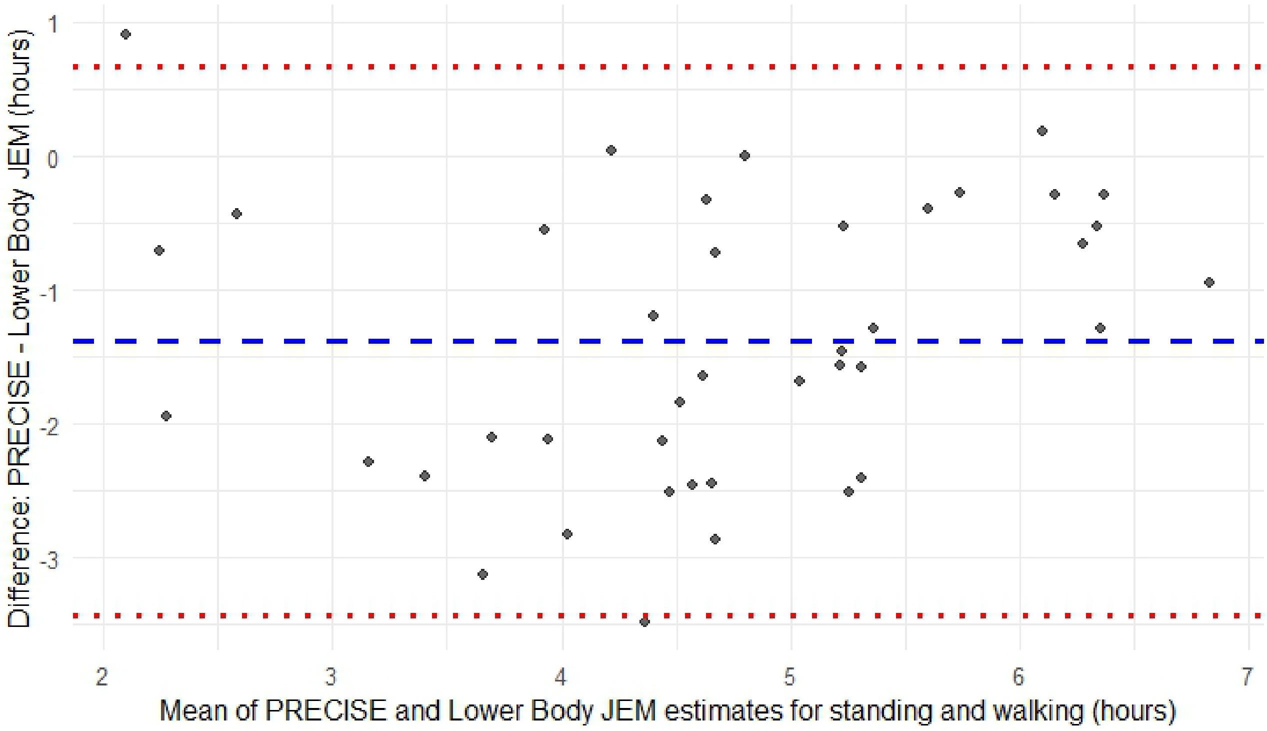

Figure S1. Bland Altman plot comparing PRECISE JEM estimates for standing and walking with the general population JEM, the Lower Body JEM (38). The compared jobs were restricted to job titles that overlapped between the two JEMs and were frequently held by female workers (selected by jobs measured in the PRECISE Cohort).

a)

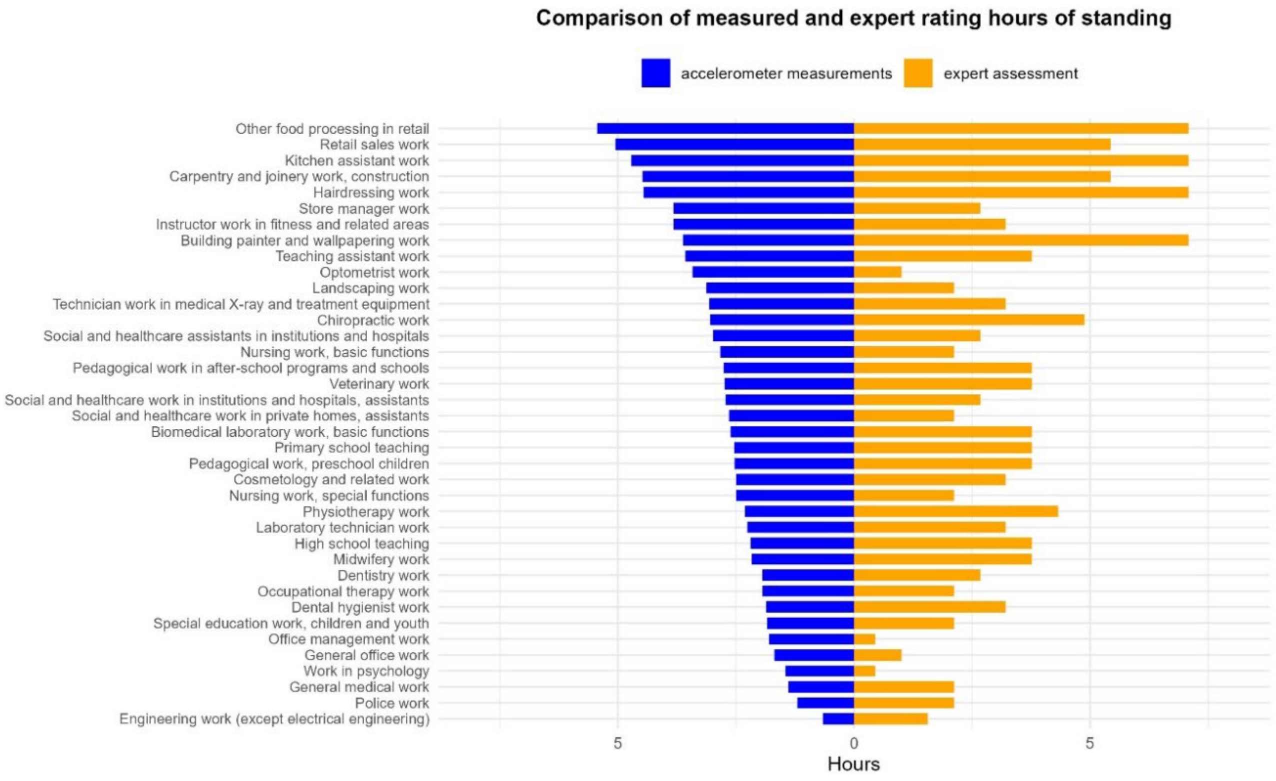

Comparison of measured and expert rating hours of standing
accelerometer measurements
expert assessment
Other food processing in retail
Retail sales work
Kitchen assistant work
Carpentry and joinery work, construction
Hairdressing work
Store manager work
Instructor work in fitness and related areas
Building painter and wallpapering work
Teaching assistant work
Optometrist work
Landscaping work
Technician work in medical X-ray and treatment equipment
Chiropractic work
Social and healthcare assistants in institutions and hospitals
Nursing work, basic functions
Pedagogical work in after-school programs and schools
Veterinary work
Social and healthcare work in institutions and hospitals, assistants
Social and healthcare work in private homes, assistants
Biomedical laboratory work, basic functions
Primary school teaching
Pedagogical work, preschool children
Cosmetology and related work
Nursing work, special functions
Physiotherapy work
Laboratory technician work
High school teaching
Midwifery work
Dentistry work
Occupational therapy work
Dental hygienist work
Special education work, children and youth
Office management work
General office work
Work in psychology
General medical work
Police work
Engineering work (except electrical engineering)
5
0
5
Hours

b)

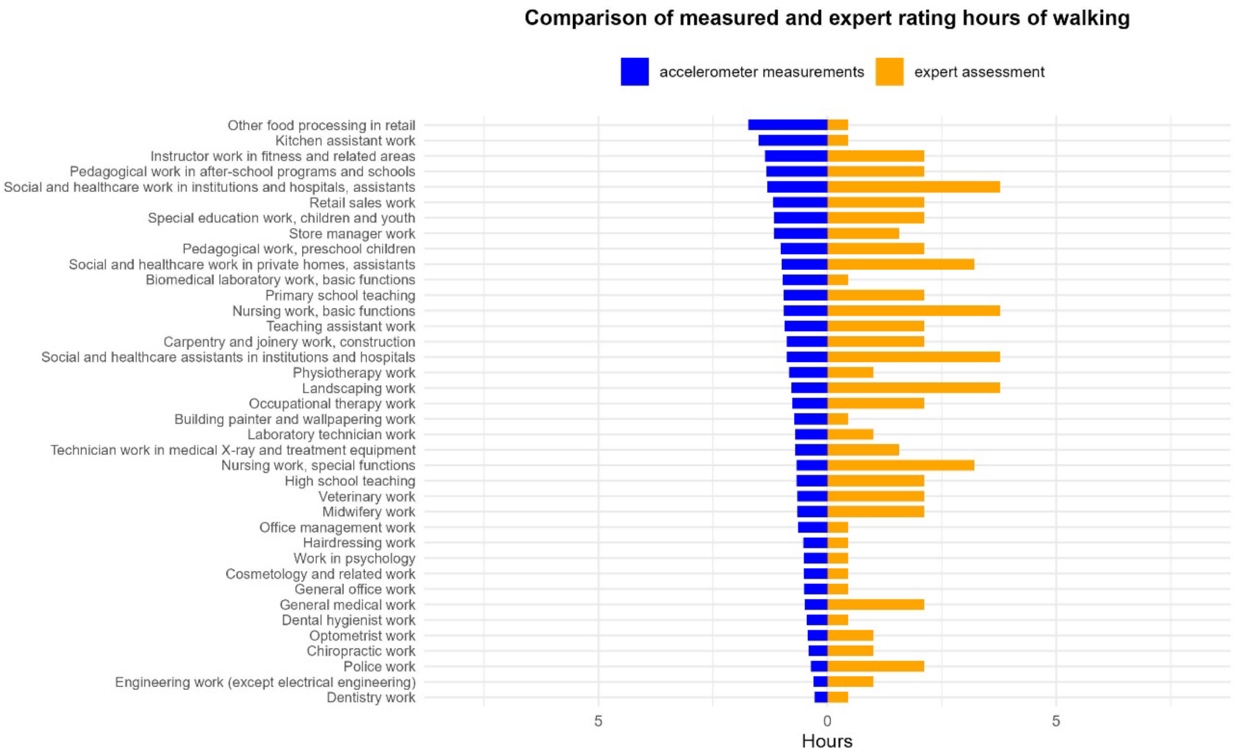

Comparison of measured and expert rating hours of walking
accelerometer measurements
expert assessment
Other food processing in retail
Kitchen assistant work
Instructor work in fitness and related areas
Pedagogical work in after-school programs and schools
Social and healthcare work in institutions and hospitals, assistants
Retail sales work
Special education work, children and youth
Store manager work
Pedagogical work, preschool children
Social and healthcare work in private homes, assistants
Biomedical laboratory work, basic functions
Primary school teaching
Nursing work, basic functions
Teaching assistant work
Carpentry and joinery work, construction
Social and healthcare assistants in institutions and hospitals
Physiotherapy work
Landscaping work
Occupational therapy work
Building painter and wallpapering work
Laboratory technician work
Technician work in medical X-ray and treatment equipment
Nursing work, special functions
High school teaching
Veterinary work
Midwifery work
Office management work
Hairdressing work
Work in psychology
Cosmetology and related work
General office work
General medical work
Dental hygienist work
Optometrist work
Chiropractic work
Police work
Engineering work (except electrical engineering)
Dentistry work
5
0
5
Hours

c)

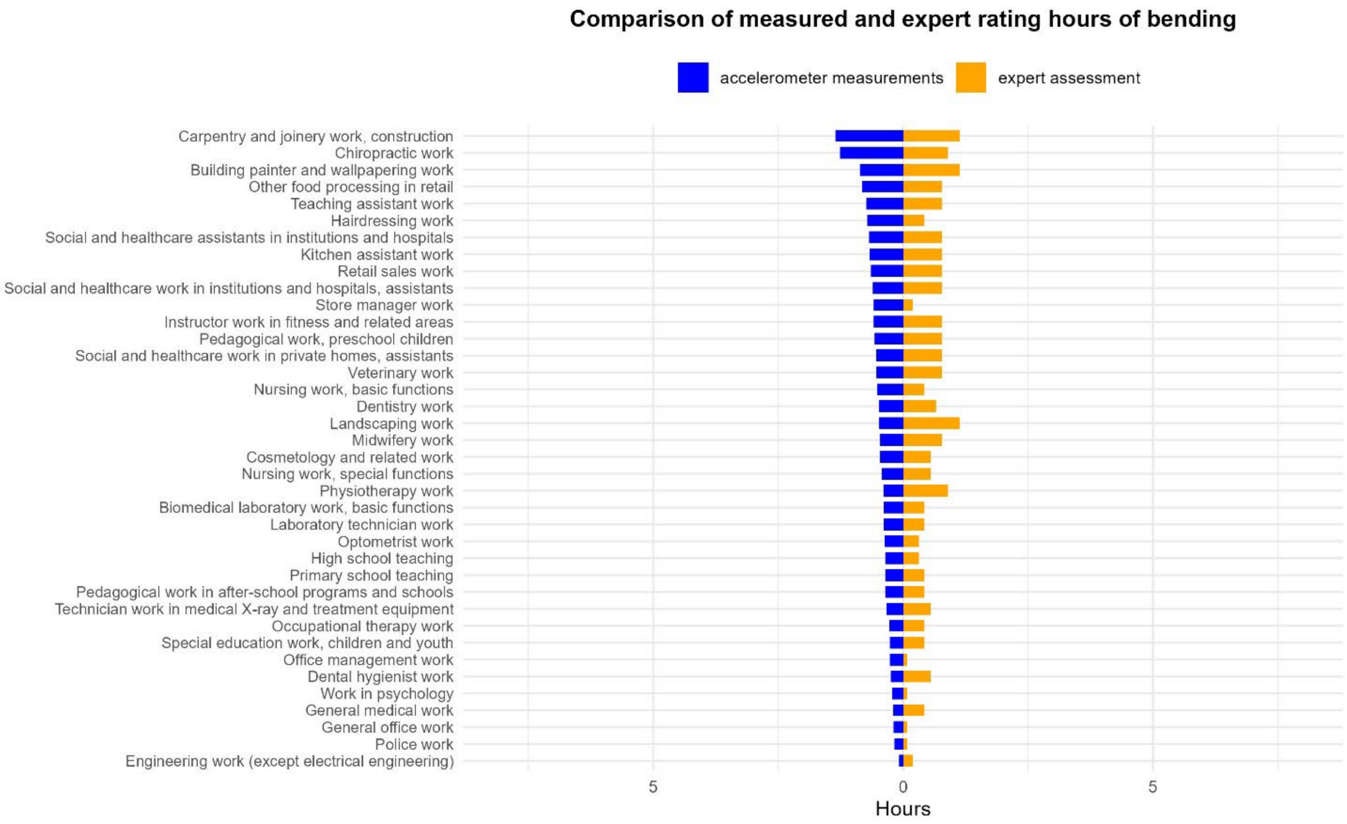
Comparison of measured and expert rating hours of bending
accelerometer measurements
expert assessment
Carpentry and joinery work, construction
Chiropractic work
Building painter and wallpapering work
Other food processing in retail
Teaching assistant work
Hairdressing work
Social and healthcare assistants in institutions and hospitals
Kitchen assistant work
Retail sales work
Social and healthcare work in institutions and hospitals, assistants
Store manager work
Instructor work in fitness and related areas
Pedagogical work, preschool children
Social and healthcare work in private homes, assistants
Veterinary work
Nursing work, basic functions
Dentistry work
Landscaping work
Midwifery work
Cosmetology and related work
Nursing work, special functions
Physiotherapy work
Biomedical laboratory work, basic functions
Laboratory technician work
Optometrist work
High school teaching
Primary school teaching
Pedagogical work in after-school programs and schools
Technician work in medical X-ray and treatment equipment
Occupational therapy work
Special education work, children and youth
Office management work
Dental hygienist work
Work in psychology
General medical work
General office work
Police work
Engineering work (except electrical engineering)
5
0
5
Hours

d)

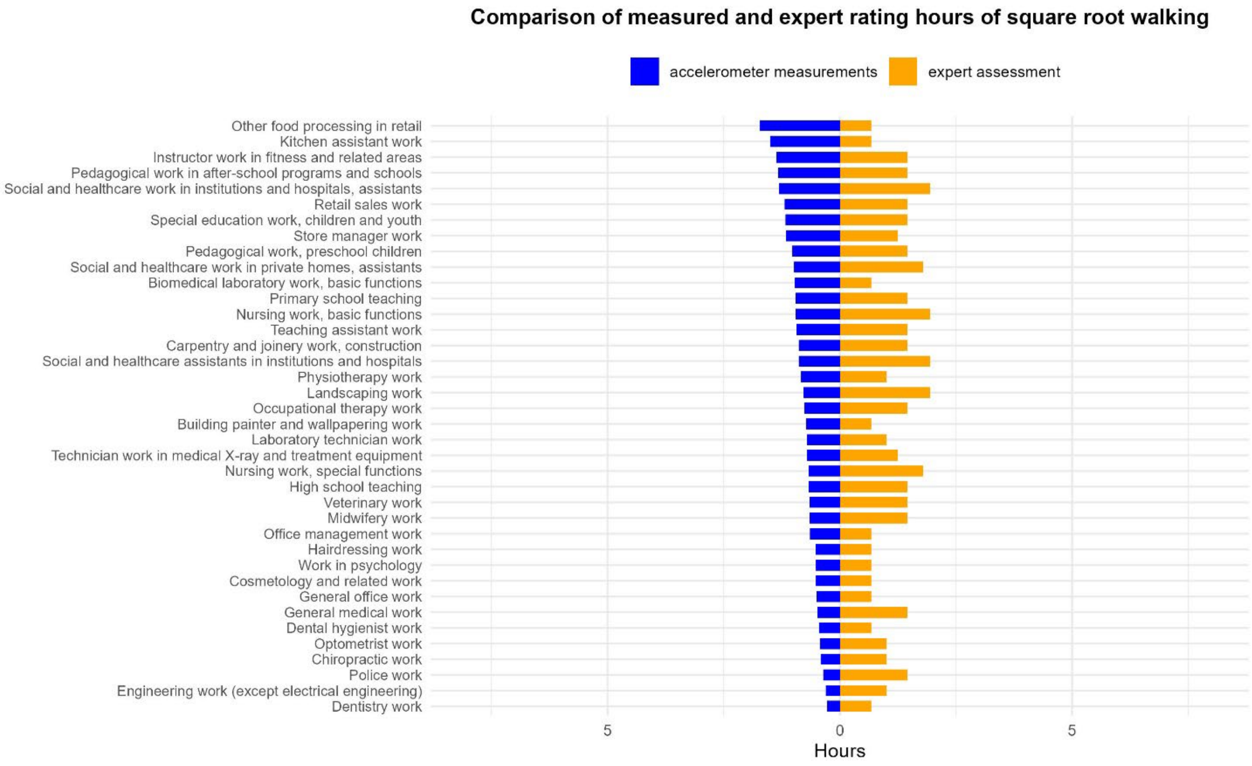
Comparison of measured and expert rating hours of square root walking
accelerometer measurements
expert assessment
Other food processing in retail
Kitchen assistant work
Instructor work in fitness and related areas
Pedagogical work in after-school programs and schools
Social and healthcare work in institutions and hospitals, assistants
Retail sales work
Special education work, children and youth
Store manager work
Pedagogical work, preschool children
Social and healthcare work in private homes, assistants
Biomedical laboratory work, basic functions
Primary school teaching
Nursing work, basic functions
Teaching assistant work
Carpentry and joinery work, construction
Social and healthcare assistants in institutions and hospitals
Physiotherapy work
Landscaping work
Occupational therapy work
Building painter and wallpapering work
Laboratory technician work
Technician work in medical X-ray and treatment equipment
Nursing work, special functions
High school teaching
Veterinary work
Midwifery work
Office management work
Hairdressing work
Work in psychology
Cosmetology and related work
General office work
General medical work
Dental hygienist work
Optometrist work
Chiropractic work
Police work
Engineering work (except electrical engineering)
Dentistry work
5
0
5
Hours

Figure S2. Mirror plots for DISCO-08 job codes with ≥3 participants, with comparisons of mean accelerometer measurements with mean expert ratings of occupational standing (a), walking (b), forward bending ≥30° (c), and the square root of walking expert assessment (d) in eight-hour times weighted averages.
